# Complete genome assemblies of two mouse subspecies reveal structural diversity of telomeres and centromeres

**DOI:** 10.1038/s41588-025-02367-z

**Published:** 2025-10-21

**Authors:** Bailey A. Francis, Landen Gozashti, Kevin Costello, Takaoki Kasahara, Olivia S. Harringmeyer, Jingtao Lilue, Tianzhen Wu, Katarzyna Zoltowska, Mohab Helmy, Tadafumi Kato, Anne Czechanski, Iraad. F. Bronner, Emma Dawson, Michael A. Quail, Anne Ferguson-Smith, Laura Reinholdt, David J. Adams, Thomas M. Keane

**Affiliations:** 1 European Molecular Biology Laboratory, European Bioinformatics Institute, Wellcome Genome Campus, Hinxton, UK; 2 Wellcome Sanger Institute, Wellcome Genome Campus, Hinxton, UK; 3 Department of Organismic and Evolutionary Biology, Department of Molecular and Cellular Biology, Museum of Comparative Zoology and Howard Hughes Medical Institute, Harvard University, Cambridge, MA USA; 4 Department of Genetics, University of Cambridge, Cambridge, UK; 5 Career Development Program and Neurodegenerative Disorders Collaboration Laboratory, RIKEN Center for Brain Science, Saitama, Japan; 6 Institute of Biology and Environmental Sciences, Carl von Ossietzky University of Oldenburg, Oldenburg, Germany; 7 Oujiang Laboratory, Wenzhou, China; 8 The Gurdon Institute, University of Cambridge, Cambridge, UK; 9 Department of Psychiatry and Behavioral Science, Juntendo University Graduate School of Medicine, Tokyo, Japan; 10 The Jackson Laboratory, Bar Harbor, ME USA; 11 School of Life Sciences, University of Nottingham, Nottingham, UK

**Keywords:** Personalized medicine, DNA sequencing

## Abstract

It has been more than 20 years since the publication of the C57BL/6J mouse reference genome, which has been a key catalyst for understanding the biology of mammalian diseases. However, the mouse reference genome still lacks telomeres and centromeres, contains 281 chromosomal sequence gaps and only partially represents many biomedically relevant loci. Here we present the first telomere-to-telomere (T2T) mouse genomes for two key inbred strains, C57BL/6J and CAST/EiJ. These T2T genomes reveal substantial variability in telomere and centromere sizes and structural organization. We thus add an additional 213 Mb of new sequence to the reference genome, which contains 517 protein-coding genes. We also examined two important but incomplete loci in the mouse genome—the pseudoautosomal region (PAR) on the sex chromosomes and KRAB zinc-finger protein loci. We identified distant locations of the PAR boundary, different copy numbers and sizes of segmental duplications and a multitude of amino acid substitution mutations in PAR genes.

## Abstract (web summary)

Telomere-to-telomere assemblies of two mouse inbred strains, C57BL/6J and CAST/EiJ, offer improvements over the current mouse reference genome by adding telomere and centromere sequences that lead to insights into variability in telomere and centromere sizes and organization, and the discovery of 225 and 355 new genes for C57BL/6J and CAST/EiJ, respectively.

## Main

Mice have been used for 100 years to model human diseases, leading to key discoveries, such as the role of the H2/MHC locus in immunity^1^, the discovery of oncogenes and tumor suppressors^2^, and the development of induced pluripotent stem cells^3^. Research using mice has provided researchers with a valuable tool for studying disease mechanisms, developing treatments, and uncovering the genetic basis of physiological processes.

In 2002, the generation and assembly of the first mouse genome for the C57BL/6J strain represented a major milestone in mouse genetics^4^. The mouse karyotype consists of 19 pairs of telocentric (TLC) autosomes and the X chromosome, with no obvious short arm, except for the Y chromosome, which is acrocentric^5,6^. TLC chromosomes are challenging to fully assemble due to the large satellite arrays near the centromere and telomere ends. The current mouse genome (GRCm39) remains incomplete due to 281 gaps distributed across every chromosome, a partial set of telomeres and no centromeres. Telomeres and centromeres are critical structural components of chromosomes, each having a unique role in maintaining chromosomal stability and integrity. High-throughput sequencing of ultralong DNA fragments (>100 kb) represents a unique opportunity to assemble fully complete telomere-to-telomere (T2T) chromosomes, as recently demonstrated by the first human T2T genome^7^.

In this study, we have used single molecule ultralong sequencing to produce the first T2T mouse genome for two inbred strains that represent two subspecies of *Mus musculus*—C57BL/6J and CAST/EiJ. Thus, we show how these T2T reference genomes are more complete than the current mouse genome (GRCm39), notably resulting in the addition of complete telomeres and centromeres for all autosomes, sequence across current gaps in GRCm39 and completing important loci such as the pseudoautosomal region (PAR) on CAST/EiJ chromosome X and two KRAB zinc-finger proteins (KZFPs) clusters.

## Results

We obtained DNA from embryonic stem cells derived from a CAST/EiJ x C57BL/6J F1 male embryo. Sequencing was performed using a combination of Pacbio HiFi (188× coverage, 42× was >15 kb read length and >Q20 Phred score) and Oxford Nanopore ultralong sequencing (70× coverage, 22× was >100 kb read length). We used a trio-based genome assembly approach using parental short reads to assign each long read to its parental haplotype (Methods). Genome assemblies were generated using both Verkko^8^ and Hifiasm^9^. We produced six distinct assemblies using both assemblers and applied a set of quality control (QC) measures to select the single best base assembly. We assessed the *k*-mer completeness to compute an overall quality value (QV) score for each assembly. Haplotype separation was evaluated by comparing *k*-mer spectra of the two haploid assemblies with those from the parental strains’ sequencing data, and by comparing them to a combined reference genome of GRCm39 and a previous PacBio long-read CAST/EiJ assembly (GCA_921999005.2). We searched for mouse canonical telomeric repeats (TTAGGG) at the end of the chromosome contigs and used the presence of telomeres as a marker for complete chromosome ends. We selected the best initial Verkko base assembly for each strain by comparing and ranking various assembly quality metrics (Supplementary Table 1), which was then improved by a round of curation (Methods), and a round of polishing that improved the base accuracy.

Several chromosomes still did not end in telomeric sequence at the centromeric end. We identified the missing telomere sequences by searching for the mouse canonical telomere repeat in the unplaced contigs. Human studies noted that large satellite arrays tend to have more similarity within a given chromosome array than between different chromosomes^10^. We applied this method in combination with long-range Hi-C mate pairs from the parental strains to identify the chromosome scaffold of greatest similarity and support from Hi-C mate pairs (Methods). This allowed us to assign all the remaining TLC sequences to a chromosome.

Table 1 provides an overview of the final C57BL/6J and CAST/EiJ T2T assemblies. The autosomes’ ungapped length for both assemblies is consistently longer than GRCm39, resulting in the addition of 208 (C57BL/6J) and 247 Mb (CAST/EiJ) of additional sequence. Base accuracy was higher in the T2T C57BL/6J than GRCm39 (QV 54.9 versus QV 54.4, respectively), and slightly lower for CAST/EiJ (QV 48.2). Mapping of parental short reads to GRCm39 and T2T C57BL/6J shows an increase of 0.7% mapped read pairs and 0.22% correctly paired reads in the C57BL/6J T2T genome (Supplementary Table 2). Additional genome QC is found in Supplementary Note 1.

**Table 1:** Genome statistics, quality metrics, repeats and gene annotation

Statistic	GRCm39	T2T C57BL/6J	T2T CAST/EiJ
Assembly			
Total ungapped length, autosomes (Gbp)	2.397	2.638	2.665
Assembly QV (Phred)	54.42	54.93	48.24
Canonical telomeres	6	38	38
Canonical telomere pairs	0	19	19
Annotation			
Protein-coding genes (BRAKER)	–	21,423	21,440
Protein-coding genes (Liftoff)	–	21,469	21,490
Repetitive bases (Mb)			
SINEs	158.954	160.028	160.736
LINEs	445.782	450.197	438.161
LTR elements	262.932	267.257	266.510
DNA elements	19.473	19.551	19.614
Unclassified	13.295	13.381	13.711
Interspersed repeats	900.437	910.416	898.734
Small RNA	1.304	1.365	1.425
Satellites	6.659	189.983	227.884
Simple repeats	61.688	64.506	67.900
Low complexity	8.677	9.002	8.956
Total repetitive bases	1879.201	2085.686	2103.631

### Chromosome structure and annotation

**Fig. 1:**
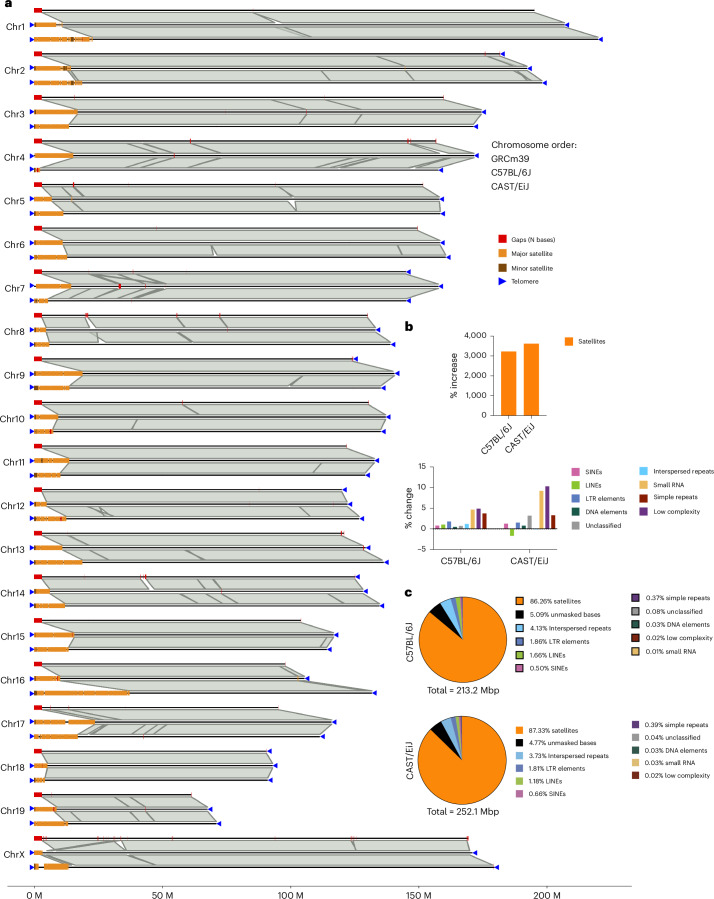
Chromosome scale synteny comparison of the mouse T2T genomes and GRCm39. **a**, Three-way synteny comparison of GRCm39 (top), T2T C57BL/6J (middle) and T2T CAST/EiJ (bottom) for all chromosomes. Gaps, centromeres (major and minor mouse satellites) and telomeres (where present) are annotated on each chromosome. Chromosome X for C57BL/6J is derived from mhaESC T2T genome^34^. **b**, Increases in satellite sequence and common repeat classes in the T2T genomes relative to GRCm39. **c**, Categorization of repeat content of new sequence in the T2T genomes with respect to GRCm39 (centromeres, telomeres and gap-filling sequences).

Figure 1 provides a synteny comparison of the GRCm39, T2T C57BL/6J and the T2T CAST/EiJ genomes that emphasizes the presence of telomeres and centromeric sequences projecting from the ends of the T2T mouse genomes. Gaps in GRCm39 that have been filled and expansions are visible, and the presence of large-scale multimegabase inversions between C57BL/6J and CAST/EiJ strains. Telomeres and centromeres are present on the ends of each T2T genome, whereas telomeres are only present on six autosomal chromosomes of GRCm39 on the noncentromeric ends. The total new sequence in the T2T genomes compared to GRCm39 is 213.2 Mb and 252.1 Mb for C57BL/6J and CAST/EiJ, respectively, and the vast majority of this consists of common repeats such as satellites and transposons (Fig. 1c and Supplementary Table 5). Total satellite sequence has been increased by more than 31-fold in both strains compared to GRCm39, along with substantial increases in all common repeat classes except LINEs in CAST/EiJ, primarily driven by the L1MdTf_I, L1MdTf_II and L1MdTf_III subclasses of LINEs (Fig. 1b and Supplementary Table 6).

Gene annotation was carried out using RNA-seq from brain, liver and various cell types for C57BL/6J and liver, brain, olfactory, spleen, testis, B cell and T cell for CAST/EiJ (Supplementary Table 7). Total protein-coding gene counts are comparable to GRCm39 (20,670 for GRCm39, 21,423 for T2T C57BL/6J and 21,440 for T2T CAST/EiJ). We also performed gene liftover from GRCm39 to the new T2T genomes using Liftoff with Ensembl 112 (Gencode 46), which annotated 21,469 and 21,490 genes for C57BL/6J and CAST/EiJ, respectively.

To identify the potential new genes, we extracted genes from the BRAKER annotation that showed no overlap with any gene from the Liftoff annotation. Considering those with at least three exons and >200 bp of coding sequence, we identified 225 and 355 new genes in C57BL/6J and CAST/EiJ, respectively (Supplementary Table 8). For C57BL/6J, new gene size varied from 552 bp to 73,352 bp and contained between 3 and 17 exons, with a corresponding total coding sequence length ranging between 201 bp and 9,288 bp. In CAST/EiJ, gene size varied from 398 bp to 74,364 bp and contained between 3 and 25 exons, with a total coding sequence ranging from 201 bp to 9,321 bp. These new genes received substantial BLAST hits to known proteins such as zinc-finger proteins.

The Liftoff annotation also identified several genes from GRCm39 that have an increased copy number in the T2T genomes (Supplementary Table 9). In total, we identified 94 and 205 genes with increased copy number compared to GRCm39 in C57BL/6J and CAST/EiJ, respectively. In C57BL/6J, *Duxf3* exhibited the highest extra copy number with 25 additional copies relative to the 3 copies annotated in GRCm39, whereas *Potefam3a* was the gene with the highest extra copy number in CAST/EiJ with 34 additional copies relative to the single copy annotated in GRCm39. Genes with increased copy number fell into a diverse range of functional categories, such as immune-associated proteins, transcription factors and signal transduction proteins. Furthermore, we identified strain-specific differences in genes with increased copy number. Notable differences include genes such as *Potefam3a* and *Sp140l1*, which displayed differences of up to 21 copies between the T2T genomes.

### Telomere and centromere structure

Telomeres and centromeres are essential for chromosome integrity, maintenance and segregation during cell division. Telomeres are repetitive nucleotide sequences at the ends of chromosomes that act as protective caps, preventing the ends of chromosomes from being recognized as DNA damage. The centromere is essential for mitotic spindle capture and checkpoint control, sister chromatid cohesion and release, and cytokinesis. GRCm39 has very limited representation of telomeres and centromeres. Historically, these sequences have been notoriously difficult to resolve due to their highly repetitive nature, and as a result, they are currently represented as gaps within the current mouse reference genome. These T2T mouse genomes dramatically improve the representation of these regions, allowing us to investigate the architecture and function of mouse telomeres and centromere sequences.

**Fig. 2:**
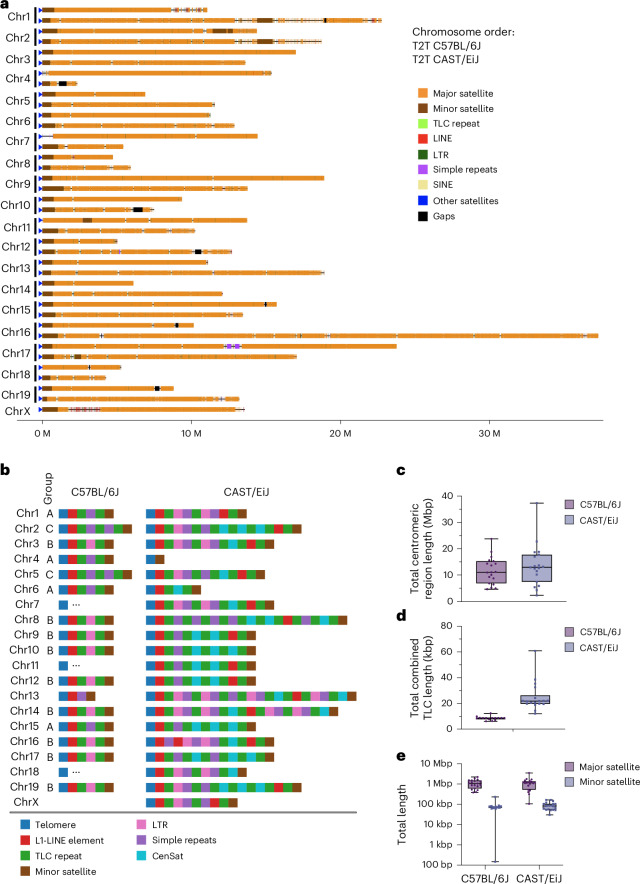
Comparison of centromeres and telomeres in the T2T genomes. **a**, Comparison of centromere chromosome ends between T2T C57BL/6J and CAST/EiJ. On each chromosome, RepeatMasker repeat classes are annotated. **b**, Structure of the subtelomeric region (TLC end) of C57BL/6J and CAST/EiJ. **c**, Centromere size distribution for T2T C57BL/6J and CAST/EiJ autosomes (*n* = 19 in both strains). Whiskers = min–max; box = IQR; line = median; dots = individual values. **d**, Size distribution of length of major and minor centromere satellite sequences per autosome (all *n* = 19 except minor satellites in C57BL/6J, which is *n* = 17). Whiskers = min–max; box = IQR; line = median; dots = individual values. **e**, Size distribution of the TLC subtelomeric repeat per autosome (*n* = 19 in both strains). Whiskers = min–max; box = IQR; line = median; dots = individual values.

Most human chromosomes are metacentric, having their centromere located in the middle of the chromosome, whereas mouse chromosomes are TLC, with their centromeres being located at the very end of the chromosome with as little as 2 kb of sequence to the telomere^11^. Figure 2a shows the location of the mouse centromeres in both strains. As expected, mouse centromeres are located directly next to the telomere, highlighting that both mouse strains have TLC chromosomes. The mouse centromere is composed of the minor satellite and is flanked by the pericentromere, which is composed of the major satellite. Together, we refer to these as the centromeric region throughout this study. The most abundant class in the centromeric region is the mouse major satellite, a 234-bp repeat monomer that has been previously reported to account for around 6–10% of the mouse genome^12,13^. In GRCm39, major satellite sequences only occupy 99.6 kb of the placed chromosomes with a median total length per chromosome of 1.8 kb, and in the C57BL/6J and CAST/EiJ T2T genomes, they account for 200.07 Mb and 223.7 Mb of the placed autosomes (7.5% and 8.4% of the genome; Supplementary Table 10). The minor satellite is the other predominant class of satellite DNA in mouse centromeres, an AT-rich 120-bp repeat monomer that has been previously reported to occupy 0.3–1 Mb per chromosome^14–16^. They are completely absent in the current mouse reference genome and are 13.07 Mb and 16.5 Mb total length in the C57BL/6J and CAST/EiJ T2T autosomes, respectively (Supplementary Table 10). We measured the sequence accuracy and the strain assignment of centromere in the genomes (Supplementary Note 2). A comparative analysis of total centromeric region size revealed substantial variability among mouse strains. The median total centromeric region size for C57BL/6J (11.1 Mb) was smaller than in CAST/EiJ (12.9 Mb). In addition, the maximum centromeric region size observed in CAST/EiJ (36.2 Mb, chromosome 16) was substantially higher than C57BL/6J (23.7 Mb, chromosome 17; Fig. 2a,c and Supplementary Table 13). The distribution of centromeric region lengths is broader in CAST/EiJ than in C57BL/6J (interquartile range 15 Mb for CAST/EiJ compared to 10 Mb for C57BL/6J), and the overall range extends from around 5 Mb to 35 Mb in CAST/EiJ compared to 5–25 Mb in C57BL/6J.

C57BL/6J TLC chromosome ends exhibit a distinct repeat organization that is shared across chromosomes^11,17^. These TLC regions are characterized by stretches of the mouse canonical telomeric repeat (TTAGGG)n, followed by subtelomeric regions composed of high-density repeat sequences. At the start of these subtelomeric regions, C57BL/6J exhibits a highly conserved L1-LINE element from the L1-MdA2 family. Following on from this LINE element, previous studies have described repeat arrays of mouse TLC repeat monomers^11,17^. These TLC arrays have been shown to be punctuated by both LTR elements and simple repeats. Finally, it has been shown that these TLC transition sequences terminate in mouse minor satellite arrays that also denote the beginning of the mouse centromeric satellite (CenSat) arrays^11,17^.

We used RepeatMasker and BLAST searches (Methods) to characterize the TLC transition sequence structure. Concordant with previous findings, we found a highly conserved L1-LINE element in 16 of 19 C57BL/6J TLC chromosome ends. However, the L1-LINE element identified in the chromosome ends is a member of the L1-MdA3 family (rather than the L1-MdA2 family observed in the earlier studies). Also consistent with the proposed model of C57BL/6J chromosome ends, we find TLC arrays immediately downstream of this L1-LINE element in 14 of 16 chromosomes. The TLC arrays in the TLC regions follow three distinct structural patterns (Fig. 2b). In the first pattern, which is observed in eight chromosomes, the C57BL/6J TLC arrays are punctuated by a conserved LTR element (RLTR17B_Mm). In the remaining two patterns, the TLC arrays are instead punctuated by AT-rich simple repeats. Of the six TLC arrays that are punctuated by simple repeats, four are punctuated by a single simple repeat, whereas the remaining two are punctuated by two simple repeats. Of the four TLC arrays with a single simple repeat, three have the same basic structure (TATA)*n* → (CATACT)*n* → (TATA)*n*, whereas the final one is composed of (CATACT)*n*. The two TLC arrays with two simple repeats have the same conserved structure, with the first repeat being (ACATAGTAT)*n* and the second repeat being (TATATGAG)*n*. In C57BL/6J, the TLC repeat is found primarily at the chromosome ends (Supplementary Fig. 1). As expected, 16 of 19 subtelomeric sequences end in minor satellite arrays.

Notable exceptions are chromosomes 7 and 11 in the C57BL/6J strain, which do not terminate in the expected manner for TLC chromosome ends. Adjacent to the telomere, there are various repetitive elements such as SINEs, LINEs and LTRs with no clear pattern. In chromosome 11, the first CenSats occur at approximately 83 kb, with an array of major satellite sequences. In this chromosome, the first instance of the minor satellite occurs at roughly 2.7 Mb with the same L1-LINE → TLC → minor satellite motif observed in other chromosomes. Chromosome 7 transit from the telomere to various LINE and LTR elements, with the first CenSats appearing at roughly 750 kb. The first instance of minor satellite sequences in this chromosome occurs at approximately 37 Mb.

The CAST/EiJ TLC chromosome end structures are highly heterogeneous with no clear shared repeat organization (Fig. 2b). Instead, CAST/EiJ showed a set of distinct repeat motifs that appear in highly variable higher-order confirmations.

However, the CAST/EiJ assembly reveals repeat motifs that are not observed in C57BL/6J TLC chromosome ends (Fig. 2b). First, CenSat repeats are observed in 18 of 20 CAST/EiJ chromosomes, whereas this repeat is totally absent in C57BL/6J chromosome ends. These CenSat repeats are always found within TLC/CenSat repeat arrays in the CAST/EiJ chromosomes. Second, repeat arrays involving LTR/TLC/simple repeats appear to be more complex in CAST/EiJ, with 12 of 20 CAST/EiJ chromosomes that show an expansion in these repeat arrays when compared to C57BL/6J (Supplementary Table 14 and Supplementary Fig. 2). Finally, 14 of 20 chromosomes in CAST/EiJ exhibit a second L1-LINE repeat. This L1-LINE repeat is always the same across CAST/EiJ chromosomes (L1MdGf_II) and appears to be a part of a CAST-specific repeat motif that leads into the centromere in 11 of these chromosomes—L1-LINE → TLC → minor satellite.

We found differences in the amount of TLC repeats between the strain assemblies. In the C57BL/6J assembly, the total amount of TLC repeat is highly conserved across chromosomes, ranging from 6.2 to 12.3 kb. Conversely, the amount of TLC in CAST/EiJ is highly variable, ranging from 12.1 to 60.5 kb. In addition, the median-combined TLC length is substantially higher in CAST/EiJ. These results are also consistent with previous experimental findings, which show substantially larger amounts of the TLC repeats in CAST/EiJ compared to C57BL/6J^11^.

### Completing the mouse reference genome

**Fig. 3:**
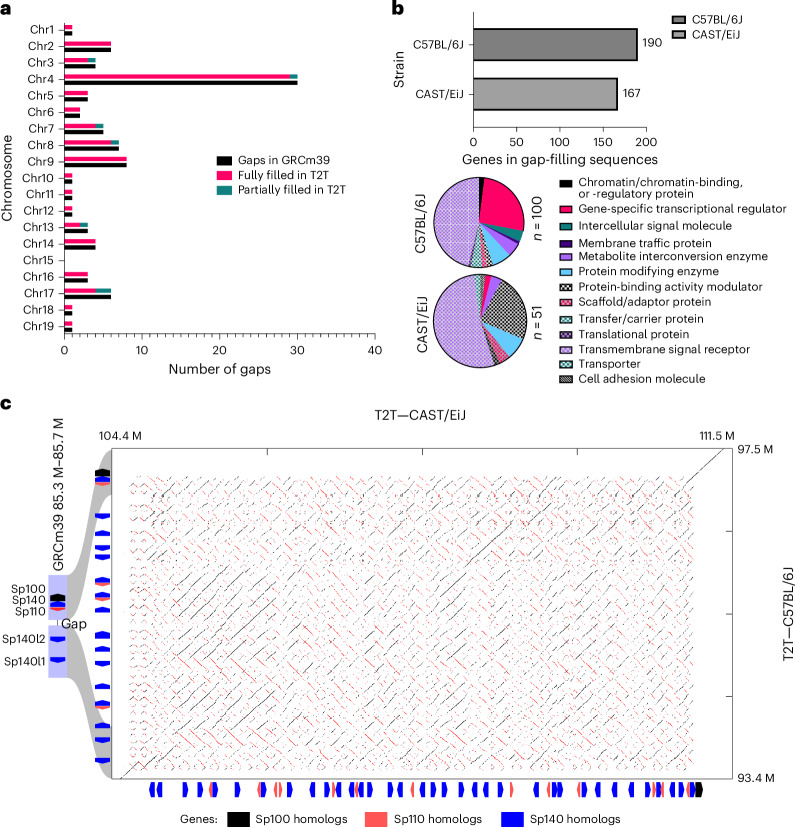
Overview, status and content of the gap regions of GRCm39 in the T2T C57BL/6J genome. **a**, Chromosome breakdown of the status of the gaps GRCm39 in the T2T C57BL/6J. **b**, Number of protein-coding genes predicted and the protein classes in the new gap-filling sequence in the T2T C57BL/6J. Genes with no PANTHER protein class were omitted from the pie charts. **c**, Example of a completely filled gap on chr1 that expanded from 50 kb in GRCm39 to 4.1 Mb in T2T C57BL/6J. A synteny comparison between T2T C57BL/6J and CAST/EiJ shows that the region is 7.1 Mb in CAST/EiJ and harbors numerous structural rearrangements. Chr, chromosome.

Despite successive efforts to fully sequence the mouse genome^18–20^, the GRCm39 reference genome contains approximately 87 autosomal sequence gaps estimated to be 5.5 Mb. Gap-filling sequences were identified in the C57BL/6J T2T assembly by mapping sequences that flank gaps in the GRCm39 assembly. The T2T C57BL/6J assembly completely spans 80 of these gaps (92%) and has partial closure of the remaining 7 gaps, introducing roughly 12.7 Mb of new sequence to the mouse genome (Fig. 3a and Supplementary Table 15). By lifting over gene annotations from GRCm39 to the T2T C57BL/6J assembly, we observe a total of 190 protein-coding genes within new gap-filling sequences (Fig. 3b and Supplementary Table 16). Functional characterization of these genes using Protein Analysis Through Evolutionary Relationships (PANTHER) revealed that the majority of these genes fall into the transmembrane signal receptor category. Other notable categories included gene-specific transcriptional regulators, transfer/carrier proteins and chromatin-associated proteins.

We found that the majority of filled gaps were consistent in size with the GRCm39 estimates (Supplementary Fig. 3). However, we observe cases where gaps substantially expand the locus such as chromosome 1 (GRCm39:1:85.32-85.3 Mb; Fig. 3c) where we have added an additional 4.1 Mb compared to a gap of 49.9 kb. Comparative analysis of this region between the T2T genomes shows that this locus is expanded by 3.0 Mb in CAST/EiJ (7.1 Mb in total) and exhibits many complex rearrangement events. Curated annotation of this gap-filling region emphasizes that it is dominated with genes that belong to the speckled protein–gene family with 21 and 49 gene family members in C57BL/6J and CAST/EiJ, respectively (Supplementary Table 17). This gene family encodes nuclear body proteins that are involved in innate and adaptive immune response and transcriptional regulation^21^. We found homologs of all members of this gene family within this selected region (*Sp100*, *Sp110*, *Sp140*), although individual gene counts differed between the strains. The most abundant speckled gene family member in this region was *Sp140* in both strains, with CAST/EiJ exhibiting 19 additional copies when compared to C57BL/6J (35 versus 16) and 9 additional copies of *Sp110* (13 versus 4).

Certain regions of the mouse genome, such as the ribosomal DNA arrays on chromosomes 12, 15, 16, 18 and 19, remain areas of ongoing investigation due to their repetitive structure. The current status of these arrays in the T2T genomes is detailed in Supplementary Fig. 4 and Supplementary Table 18.

### PAR

The PAR, which is shared by the X and Y chromosomes and located at the ends of them, contains numerous repeated sequences^22^ and a high GC content^23^, likely resulting from a high recombination frequency in this region (>100 times the genome average)^24,25^—one of the most challenging euchromatic regions to sequence and, as a result, only partial PAR sequences were included in GRCm39. In 2012, a ~430-kb shift in the PAR boundary (PAB) in *M. musculus castaneus* was identified, which contributed to a marked lineage-specific increase in sequence divergence within *Mid1* (ref. ^26^). In this study, we assembled the CAST/EiJ X chromosome PAR sequence, except for a large SD structure that could not be resolved. We compared this to the C57BL/6J X chromosome PAR that was produced from a prior assembly of the same strain^27^.

**Fig. 4:**
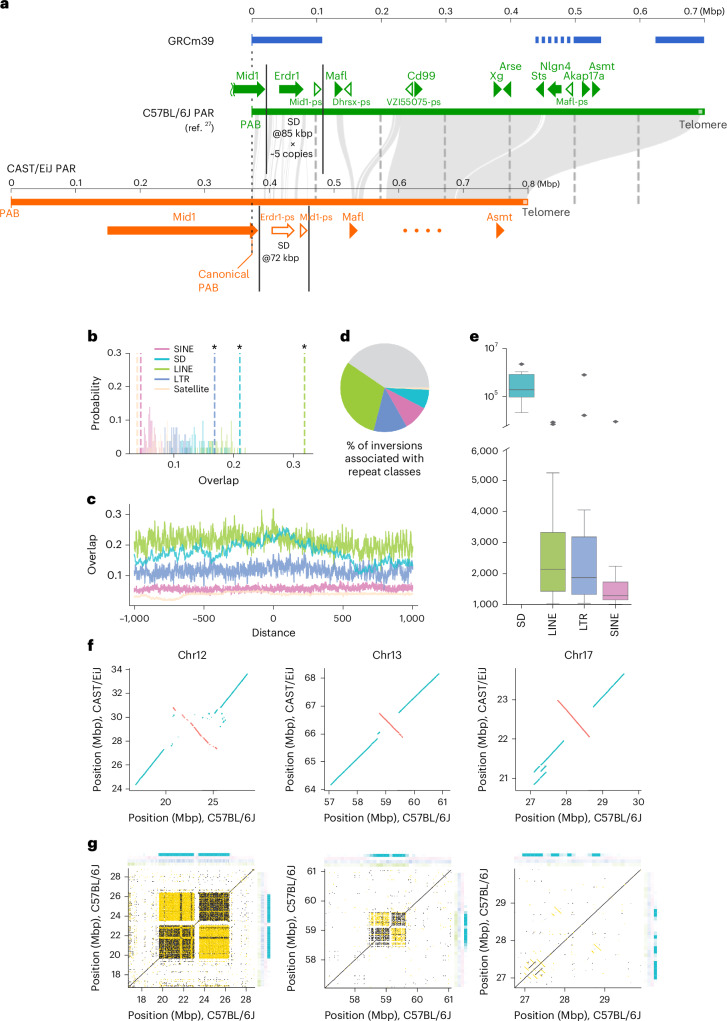
PAR and large-scale inversions. **a**, A comparison of the mouse PAR locus among GRCm39, C57BL/6J and T2T CAST/EiJ genomes. The PAB refers to the pseudoautosomal boundary, which is different in each strain. Tick marks in the synteny plot denote 100-kb intervals. **b**, Expected overlap at inversion breakpoints from 1,000 random resampled permutations (histograms) compared to observed overlap (dotted lines) for different repeats. Asterisks indicate substantial enrichment (FDR corrected two-sided permutation test; *Q* < 0.001). **c**, Repeat overlap (proportion of nucleotides attributed to a given repeat) calculated across 1-kb windows relative to inversion breakpoints in the GRCm39 genome (centered on zero) for various repeat types. **d**, Proportion of inversions associated with NAHR between different repeats. **e**, Length distributions for inversions associated with each considered genomic repeat. The center of each box displays the median. The lower and upper bounds of each box represent the 25th percentile and 75th percentile, respectively. Whiskers extend from the bounds of the box to the minimum and maximum values within 1.5× interquartile ranges of the lower and upper quartiles. Any data point outside this range is considered an outlier and plotted individually. **f**, Dotplots for selected inversion regions generated from whole-genome alignments between T2T C57BL/6J and CAST/EiJ. Collinear and inverted alignments are plotted in blue and red, respectively. **g**, Dotplots generated from self-versus-self alignments of selected inversion breakpoint regions in the B6 genome. Collinear and inverted alignments are plotted in black and gold, respectively. Heatmaps show repeat density calculated across 10 kb. Heatmap colors correspond to repeat colors used in **b**–**d**.

In the mouse PAR, ten genes (four of which were new) and four pseudogenes were identified (Fig. 4a), which show synteny with the human PAR1 (Supplementary Fig. 5) and differences in large repeat units and copy numbers (Supplementary Note 3).

### Inversions

Inversions are thought to have an important role in speciation and local adaptation by suppressing recombination; for example, a heterozygous inversion can drastically increase linkage disequilibrium between the loci it carries^28^. In mice, inversions have been associated with skeletal abnormalities^29^, impaired growth of palate shelves^30^ and dwarfism^31^. Comparison of T2T assemblies revealed multiple large inversions between mouse strains, shedding light on their origins. Chromosomal inversions are genomic structural rearrangements in which a region of a chromosome is reversed between haplotypes. Inversions can play substantial roles in evolution, but are challenging to study because their breakpoints often occur in highly repetitive genomic regions that are poorly assembled^32,33^. By comparing T2T assemblies, we identified 133 (>1 kb) inversions among C57BL/6J, CAST/EiJ and mhaESC^34^ (Supplementary Table 19). Inversions are often formed through nonallelic homologous recombination (NAHR) between genomic repeats^35,36^. Thus, to study the origins of inversions in house mice, we investigated repeat content at inversion breakpoints. We performed permutation tests comparing repeat overlap at inversion breakpoints to expectations from randomization, as well as surrounding regions. Inversion breakpoints show enrichment for SDs as well as for LINE and LTR retrotransposons, suggesting that these repeats may have roles in inversion formation (Fig. 4b; permutation test, *n* = 100, *P* < 0.001 for all). LINE and LTR enrichment is highly localized and decays rapidly with distance from inversion breakpoints, consistent with the expected size of these TEs (~200 bp to ~8 kb; Fig. 4c). In contrast, SD enrichment extends hundreds of kilobases from inversion breakpoints, suggesting that inversions frequently inhabit complex SD-rich genomic regions (Fig. 4c). To quantitatively estimate the number of inversions associated with different genomic repeats, we searched for patterns consistent with NAHR in which inversions are flanked by homologous repeats at both breakpoints^36,37^. Overall, ~60% of inversions show patterns consistent with NAHR, of these ~50% are associated with LINEs, ~21% with LTRs, ~15% with SINEs and ~11% with SDs, respectively (Fig. 4d). Notably, although retrotransposons appear to have facilitated the majority of NAHR-mediated inversions, SD-associated inversions are substantially longer (MWU, *P* < 0.001; Fig. 4e). Furthermore, larger SDs are associated with larger inversions (Kendall’s *τ* = 0.63, *P* = 0.0007; Supplementary Fig. 6). These results are consistent with observations in humans and deer mice, suggesting that larger repeats are required to facilitate larger structural rearrangements^37^. We then focused on the largest inversions, because longer inversions have more profound effects on genome structure and recombination. We identified several large inversions that are greater than 1 Mb in length (Fig. 4f). Large inversions on both chromosomes 12 and 13 involve highly complex genomic regions that primarily contain SDs (Fig. 4g). A large inversion on chromosome 17 shows long inverted repeats at its breakpoints, suggesting that it arose through NAHR (Fig. 4g).

### KRAB zinc-finger loci

T2T assemblies have greatly improved the coverage of KZFPs. KZFPs are one of the largest families of transcription factors in vertebrate genomes^1^. KZFPs preferentially bind transposable elements (TEs) to recruit repressive epigenetic modifications^38–40^. KZFPs are highly homologous and have been proposed to evolve through SDs, resulting in a large number of KZFPs existing in clusters within mammalian genomes^41,42^. These elements are highly polymorphic in both between species^38,39^ and between strains of mice, where strain-specific epigenetic modifiers have been identified in KZFP clusters^43,44^. These loci are incomplete in GRCm39, limiting the ability to effectively profile the evolution and divergence of mouse KZFPs.

**Fig. 5:**
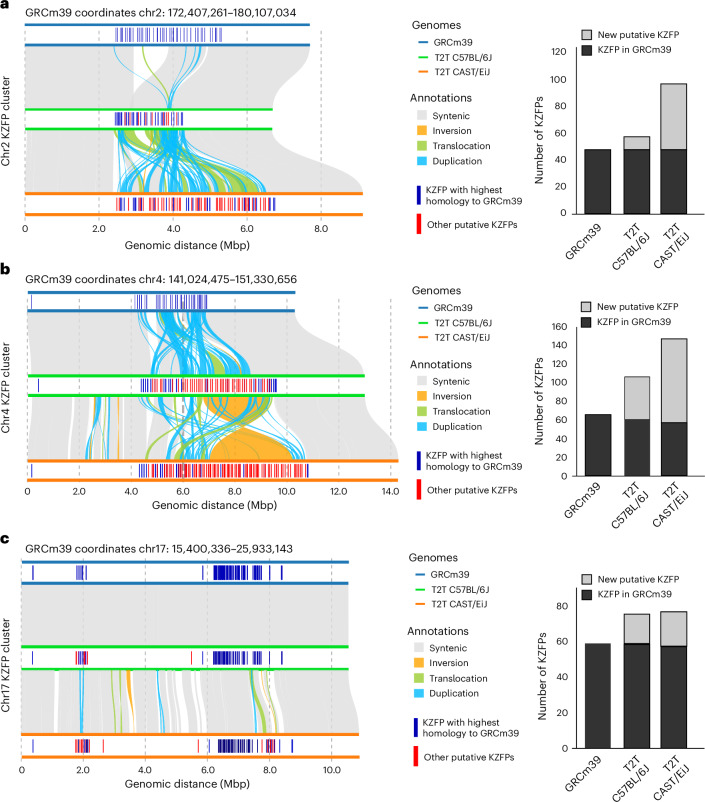
Comparison of the KRAB zinc-finger protein clusters between the GRCm39, T2T C57BL/6J and CAST/EiJ genomes. **a**–**c**, Synteny plots for clusters on chr2 (**a**), chr4 (**b**) and chr17 (**c**) are provided. Bar plots (right) give the number of KZFP proteins in each cluster per strain.

The distal arms of chromosomes 2 and 4 contain two of the largest clusters of KZFPs that are incomplete in GRCm39. The T2T assemblies have resolved the sequence of these KZFP clusters. For example, more than 48 new putative KZFPs in the C57BL/6J T2T genome have been identified and large-scale structural variations in KZFP clusters (Fig. 5a–c and Supplementary Note 4).

## Discussion

It has been more than 20 years since the first version of the mouse genome was released. The current version of the reference genome, GRCm39, remains incomplete and lacks sequence in many important loci. The present study marks a major milestone toward the complete and accurate characterization of chromosomes in the mouse. We used the latest ultralong sequencing technologies to produce T2T mouse reference genomes for two highly used strains (C57BL/6J and CAST/EiJ), bringing to three the number of T2T mouse genomes, including mhaESC^34^ (Supplementary Table 20). We add 213 Mb of sequence to the mouse reference genome containing an estimated 517 protein-coding genes, providing sequence for all gaps in the current mouse reference. These genomes provide a comprehensive set of centromeres and telomeres for all autosomes, and we performed a detailed comparative analysis of mouse telomeres and centromeres between two subspecies, revealing differences in both size and structure. Important loci such as the PAR locus, KZFP loci and gap regions enriched for immunity loci can now be studied in much more detail as the complete sequence will accelerate functional experiments and evolutionary analysis.

Inversions are copy-neutral structural variants (SVs) that have the potential to disrupt the regulatory interactions of genes and have proven to be the most challenging form of SVs to accurately detect using array- and sequencing-based approaches. We have demonstrated how complete T2T genomes have enabled the generation of a comprehensive set of inversions between the C57BL/6J and CAST/EiJ strains, revealing several megabase-scale inversions. The largest inversions are densely flanked by SDs, a feature that has been identified in distant species such as deer mice and humans, pointing toward a universal mechanism.

We highlighted a number of important loci that were missing or partially complete in GRCm39. For example, the KZFP clusters are known to be linked to strain-specific epigenetic outcomes; being able to annotate and properly profile the KZFPs across strains of mice is essential to our understanding of how the epigenetic landscape is established and evolved. From T2T genome assemblies, we can annotate and resolve previously unresolved genomic elements, which are among the most divergent between mouse strains. This allows us to uncover the mechanisms behind how these elements evolve and drive divergent regulation of mammalian genomes.

This study represents a major milestone for mouse genetics that enables future functional studies in the incomplete regions of the mouse genome. Inbred and outbred hybrid mouse populations, such as the Diversity Outbred Cross^45^ and Collaborative Cross^46^, are now being used to fine-map a plethora of newly discovered QTL loci; the addition of complete sequence for key loci in two founder strains will accelerate this process. The future expansion of T2T reference genomes to include additional strains will form the basis for the mouse pangenome to fully represent the genetic diversity of mice.

## Methods

### Mice

Mouse embryonic stem cells were derived from 3.5 dpc F1 embryos from a cross between CAST/EiJ (RRID:IMSR_JAX:000928) dams and C57BL/6J (RRID:IMSR_JAX:000664) sires. Derivation and characterization (including mycoplasma testing, single nucleotide polymorphism genotyping, pluripotency marker expression, chromosome counting and germline testing) of the mESCs were previously described^47^. One male line (CASTB6-9) with a >70% euploid karyotype was selected for sequencing. CASTB6-9 was cultured as previously described^47^, dissociated, washed in PBS and then pelleted before being flash-frozen in liquid nitrogen and stored at −80 °C. Thus, 5 × 10^6^ and 1 × 10^6^ cell aliquots were shipped on dry ice to the Sanger Institute for sequencing. For the digital PCR measurement of PAR regions, C57BL/6J mice (RRID:IMSR_JAX:000664) were used. All procedures involving laboratory mice were approved by the Institutional Animal Care and Use Committees of the Jackson Laboratory (under Animal Use Summary, 20030) and RIKEN (approval W2021-2-042(2)).

### DNA and sequencing

High-molecular-weight DNA was extracted from 1 million (PacBio) to 5 million (Oxford Nanopore - ONT) cell pellets using the Monarch T3050 kit and protocol (New England Biolabs). For ONT ultralong, recommended protocol adjustments were carried out. DNA QC was performed using FemtoPulse, Qubit measurement taken from the top, middle and bottom of the extractions and homogenization of extracted DNA by gently pipetting with a wide bore pipette tip. Qubit measurements from the top, middle and bottom of the tube were repeated until values were similar. Library preparation for PacBio sequencing uses template preparation kit 2.0. Sequencing on PacBio Sequel IIe with SMRT cell 8M uses binding kit 2.2 and sequencing kit 2.0a (six SMRT cells were run in total). ONT library preparation was performed with ONT’s UL sequencing kit (ULK001), followed by sequencing on PromethION 10.4.1 (runs were monitored to perform multiple nuclease flushes and reload more library).

### Genome assembly

Our C57BL/6J and CAST/EiJ assemblies were generated using a combination of haplotype-aware, T2T-capable genome assembly approaches—(1) Verkko (v1.3.1) and (2) Hifiasm (v0.19.5). We generated multiple assemblies using different combinations of read quality and read length subsets. All assemblies were executed in trio-binning mode, using both HiFi and ONT reads, and *k*-mer databases generated from strain-specific Illumina short reads as input. Parental *k*-mer databases were generated by Merqury (v1.3)^48^ and Yak (v0.1-r66-dirty, https://github.com/lh3/yak) for Verkko and Hifiasm, respectively, following each assembler’s recommended guidelines. For each assembly, this method produced a set of haplotype-separated contigs for each strain that were ordered and oriented into chromosome scaffolds with RagTag (v2.1.0)^49^, using each strain’s respective reference genome (C57BL/6J: GCA_000001635.9; CAST/EiJ: GCA_921999005.2) as an anchor.

### Assembly evaluation

Assemblies were evaluated and compared to identify the best set of chromosome scaffolds to identify each strain’s respective base genome assembly. Merqury (v1.3) was used to assess the *k*-mer completeness. In the context of the mouse T2T assemblies, haplotype separation refers to separating into a C57BL/6J assembly and a CAST/EiJ assembly. Haplotype separation in each of our assemblies was evaluated using the ‘trioeval’ command in Yak (v0.1-r66-dirty, https://github.com/lh3/yak) that compares each haplotype-separated assembly to *k*-mer spectrums generated from parental Illumina reads. These comparisons are used to calculate switch error and Hamming error rates. Haplotype separation was further evaluated by aligning haplotype-separated contigs to a combined GRCm39 and a pure CAST/EiJ long-read reference genome (GCA_921999005.2) with minimap2 (v2.24-r1122)^50^. Mouse canonical telomeric repeats (TTAGGGn) were detected in each of our assemblies using the ‘telo’ command in seqtk (v1.4-r130-dirty, https://github.com/lh3/seqtk) that searches for telomeric repeats at the end of each sequence within a FASTA file to evaluate chromosomes properly terminated in canonical telomeric repeat. Finally, we also compared the number of hybrid SV calls reported by Sniffles (v2.0.7)^51^ (default parameters), using both HiFi and ONT read alignment and used these counts as a measure of assembly accuracy.

We used the information generated above to rank each of our candidate mouse strain assemblies against each other. We chose the assembly that ranked the highest across our chosen criteria to become our base C57BL/6J and CAST/EiJ chromosomes (Supplementary Table 1). Both selected assemblies for each strain were generated using Verkko. For each strain, chromosome-to-chromosome alignment comparisons were generated between our assemblies with Winnowmap (v2.03)^52^ (default parameters). When we identified cases where a given telomeric region was missing in the base assembly chromosomes but was present in a given secondary assembly, we incorporated the corresponding sequences from the secondary assembly into the base chromosomal assembly using seqtk (v1.4-r130-dirty, https://github.com/lh3/seqtk). All assembly changes were supported by both HiFi and ONT read alignments generated by Winnowmap, where multiple reads spanned our integration boundary.

### Assembly polishing

We performed a hybrid HiFi and ONT read-based error correction pipeline, previously outlined in ref. ^53^. Sniffles was used to call SVs in our assemblies using both HiFi and ONT read alignments (v2.0.7)^51^, and the insertion and deletion sequences from these SV calls were then polished using Iris (v1.0.4, https://github.com/mkirsche/Iris). Next, Jasmine (v1.1.5, 10.1101/2021.05.27.445886) was used to merge our independent HiFi and ONT call sets to identify all variants that were observed using both sequencing technologies. Finally, we filtered these shared variants using Merfin^54^ and incorporated these SV corrections into our final assemblies with bcftools^55^. All tools were run using their default parameters. This polishing process improved the base accuracy of both assemblies—C57BL/6J (47.7 to 54.9 QV) and CAST/EiJ (44.4 to 44.6 QV).

### Assembly curation

Hi-C reads were mapped to the T2T genomes following the Arima Hi-C mapping pipeline (https://github.com/ArimaGenomics/mapping_pipeline). We generated and visualized a Hi-C contact map using PretextMap (v0.1.9, https://github.com/sanger-tol/PretextMap) and PretextView (v0.2.5, https://github.com/sanger-tol/PretextView), which was used to manually curate our chromosomes. After this process, several mouse chromosomes still lacked telomeric sequences on their centromeric ends. We identified these ‘missing’ telomere sequences by searching for the mouse canonical telomere repeat in our assemblies’ unplaced contigs using the ‘telo’ command in seqtk (v1.4-r130-dirty, https://github.com/lh3/seqtk). RepeatMasker analysis (for details on how RepeatMasker analysis was performed, see Repetitive sequence annotation) revealed that many of these contigs also contained centromere repeats, supporting their placement in the ‘missing’ regions in our assemblies. To assign these TLC contigs to the correct chromosome, we used a mapping-based approach using MashMap (v3.1.1)^56^. It has previously been noted in human studies that large satellite arrays tend to have more similarity within a given chromosome array than between different chromosomes^10^. Therefore, we used MashMap (--pi 95 -f one-to-one) to map our unplaced TLC sequences against our chromosome-assigned scaffolds and quantified their sequence similarity to each chromosome scaffold by computing the cumulative alignment lengths per chromosome. This was used to identify the chromosome scaffold with the highest amount of similarity. We also quantified the amount of Hi-C read pairs with one mate on a given unplaced TLC sequence and a given chromosome scaffold to provide supporting evidence of linkage to a particular chromosome. Finally, we assigned all remaining TLC sequences to a chromosome, introducing a model gap of 100 bp between the contig and the chromosome using seqtk (v1.4-r130-dirty, https://github.com/lh3/seqtk). The result of this process now meant that all of our mouse chromosomes ended in mouse canonical telomere repeat on both ends. The final chromosomes are available under accessions GCA_964188545.1 (CAST/EiJ) and GCA_964188535.1 (C57BL/6J).

### Gene prediction and annotation

We used BRAKER3 (v3.0.3)^57^ (default parameters) to predict protein-coding gene structures in our assemblies using both RNA-seq and protein evidence to train the gene prediction pipeline. RNA-seq data were acquired from the ENCODE portal^58^ and public databases (Supplementary Table 4). RNA-seq reads were then aligned to each strain’s respective genome using STAR (v2.7.10b)^59^. For protein evidence, we used the Vertebrata database acquired from OrthoDB^60^.

In addition to our BRAKER3 de novo gene prediction, we also produced an annotation transferring genes from GRCm39 to our new T2T assemblies using Liftoff^61^ (v1.6.3) using the following arguments: -copies -sc 0.95 -polish -exclude_partial.

### Repetitive sequence annotation

We used RepeatMasker (v4.1.5, http://www.repeatmasker.org) with the default Dfam repetitive element library in ‘mus musculus’ mode to identify and annotate repetitive elements in our new C57BL/6J and CAST/EiJ genomes. To validate and refine our repeat annotations, we further supported our RepeatMasker annotations with targeted BLAST searches using C57BL/6J reference sequences for the minor satellite and TLC repeats, and the ribosomal DNA repeating unit^11,62–64^.

#### Identification of new genes

We extracted genes from each BRAKER annotation that exhibited no overlap with any gene from the Liftoff annotation using bcftools (v2.31.0), subcommand intersect -v. The output GFF3 file was then filtered to include only BRAKER gene entries that had ≥3 exons and ≥200 bp of coding sequence. The protein sequences for these filtered genes were then used as query sequences for a BLASTp search against all C57BL/6J proteins in the Ensembl genome browser^65^. Each BRAKER gene was assigned a top BLAST hit from this search to infer its potential function (Supplementary Table 5).

#### Identification of genes with an increased copy number

Genes with an increased copy number were identified using the ‘extra_copy_number = XX’ tag in the GFF output from Liftoff (v1.6.3). In brief, Liftoff searches for additional copies of genes from the GRCm39 annotation file in the T2T genomes. With the ‘-copies’ and ‘-sc 0.95’ arguments, only additional gene copies with at least 95% of the coding sequence aligned were classified as duplications. To refine the final gene list, the ‘--exclude_partial’ parameter was used to filter out partial and fragmented gene copies. To establish the corresponding gene copy number in the GRCm39 reference genome and minimize the effects of potential gene misannotations, we re-annotated GRCm39 using Liftoff with its corresponding gene annotation file (command: liftoff -p 24 -copies -sc 0.95 -polish -exclude_partial -g Mus_musculus.GRCm39.112.chr.gff3 -dir m39_gff3_e112 -o liftoff.GRCm39.ensembl_112.gff3 Mus_musculus.GRCm39.dna.toplevel.fa). Gene copy numbers identified in the T2T assembly were then quantified relative to this refined GRCm39 annotation.

#### Centromere accuracy

We aligned our F1 ONT reads onto a combined reference genome consisting of both T2T genomes using minimap2 (v2.17-r941). We converted the PAF files to BED and generated per-base coverage using bedtools ‘genomecov’ subcommand. We identified positions in the centromere regions where the coverage was greater than twice the sequencing coverage (greater than 70×). The resulting coordinates of high coverage in the centromere regions are provided in Supplementary Table 12.

#### Centromere strain assignment

We aligned the mhaESC B6J ONT reads onto a combined reference genome consisting of both T2T genomes using minimap2 (v2.17-r941). For each read, we selected the hit with the highest alignment score. In Supplementary Table 11, we provide the regions in the CAST/EiJ centromeres that have greater than 10x coverage of mhaESC reads.

#### PANTHER protein classification

We used the PANTHER classification system (v19.0)^66^ to assign protein classes to genes of interest within our Liftoff annotations (Supplementary Table 6). For a given Liftoff gene, this was achieved by using its associated Ensembl gene ID, lifted over from GRCm39 annotation, into the PANTHER web-based server (https://pantherdb.org/).

### Identification of gap-filling sequences

We implemented an alignment-based approach using the repeat-sensitive alignment software Winnowmap (v2.03)^52^. Gap positions in GRCm39 were extracted from the AGP file from NCBI (https://hgdownload.soe.ucsc.edu/goldenPath/mm39/bigZips/mm39.agp.gz). This file was filtered to exclude all model gaps for the centromeres, telomeres and short arm. We extracted the flanking sequences (ranging from 50 kb to 200 kb) of the remaining 87 gaps in GRCm39 autosomes and aligned them to our new C57BL/6J assembly with Winnowmap, following the tool’s recommended guidelines for mapping WGS reads, as they are of comparable size to the flanking sequences. Gap-filling sequences were then inferred as the sequence between each gap’s left and right flanking sequence alignments. Using these gap-filling sequences, we characterized gaps as follows: completely filled (new sequence added with no gap bases remaining); partially filled (new sequence added with some gap bases still remaining) or not filled (only one/no flanking sequence alignment or no non-N bases added).

### PAR assembly using PacBio walking method

PacBio HiFi reads from C57BL/6J genomic DNA (SRR11606870)^67^ were mapped to known ‘seed’ sequences, that is, any of the exons of *Asmt*, *Akap17a*, *Mafl-ps*, *Nlgn4*, *Sts*, *Arse*, *Mafl*, *Erdr1*, *Mid1* and *Gm52481* genes, using minimap2 (v2.17-r941) with the parameters -k 27 -w 18 -m 99. The alignments were processed using samtools (v1.1) and visualized with IGV (v2.8.13). We manually selected reads that were identical (except for obvious mutations or polymorphisms in the seed sequence) to the seed sequence over 4 kb and assembled them using CAP3 version date: 21 December 2007) with the default parameters. If more than two contigs were generated, the contig consisting of the largest number of reads was used as the representative. To visualize the hallmark of the contig sequence, we created a dotplot view of self-similarity using web YASS (https://bioinfo.lifl.fr/yass/yass.php) or local YASS (v1.15) with the default parameters. We compared the self-similarity view of the contig with that of the seed sequence and confirmed that the walking was proceeding correctly. When the self-similarity view of the contig that we took as representative was obviously different from that of the seed sequence, we used another contig as an alternative representative. Next, the representative contig and the seed sequence were compared using the BLASTN 2 sequences program (default parameters) and then manually merged. Basically, the contig sequence was connected to the seed sequence near the center where these sequences overlapped. A 10-kb sequence from the end of the merged sequence was used as the new seed for the next round of walking. Each round of walking yielded a new sequence of 3.5–12.5 kb (~9.8 kb on average).

### PAR digital PCR

Genome DNA was extracted from liver, brain or tail chip of C57BL/6J mice using Monarch genomic DNA purification kit (T3010S, New England Biolabs) and digested by *Pst* I (R3140S, New England Biolabs). After heat inactivation (60 °C for 15 min) and dilution with water (5 ng µl^−1^), we performed digital PCR using the QuantStudio 3D system (Thermo Fisher Scientific) according to the manufacturer’s procedure. We used TaqMan Copy Number Reference Assay, mouse, Tfrc (4458366) to count the chromosome 16 (two copies of each of the diploid genome) and Custom TaqMan Copy Number Assays (mMid1Ex5 and mMid1Ex7), which targeted the exons 5 and 7 of *Mid1* gene, designed by the TaqMan Custom Design Assay Tool (Thermo Fisher Scientific). The sequences of the primers and probes are shown in Supplementary Table 21.

The copy numbers of the SD obtained by using mMid1Ex5 and mMid1Ex7 primer assays were the same. The copy numbers in DNA samples extracted from the liver, brain and tail tip of the same individual were the same, indicating that the copy number did not change during ontogeny.

### Inversions methods

We first aligned C57BL/6J and CAST/EiJ T2T genomes using minimap2 (v2.21) with flags-a --eqx -x asm5 --cs -r2k (ref. ^50^). We sorted and indexed resulting BAM files using samtools (v1.10)^68^ and called inversions using SyRI (v1.6.3)^69^, which uses alignment of syntenic regions to accurately detect structural rearrangements. SyRI performs particularly well in identifying large balanced SVs, such as inversions, from whole-genome alignments compared to other available tools. Due to the challenges with systematically calling balanced SVs in highly repetitive regions, we filtered out inversions primarily composed of simple repeats and satellites and manually inspected dotplots to filter out spurious calls^37,70,71^. We also filtered out erroneous balanced inversion calls likely caused by twin-priming during L1 retrotransposition by removing inversions covered ≥95% by L1 models^72^. After filtering, we were left with 131 inversions larger than 1 kb (Supplementary Table 12).

To investigate the genomic mechanisms underlying inversions in house mice, we explored repeats at inversion breakpoint regions. We first performed permutation tests for enrichment of repeats in inversion breakpoint regions for the following five types of repeats: LINEs, SINEs, LTR retrotransposons, satellites and SDs. Specifically, we obtained the 1-kb flanking regions surrounding each inversion breakpoint using bedtools flank (v2.29.1)^73^. We then assessed various metrics of repeat composition in these regions, comparing them to expectations derived from 1,000 randomly resampled permutations using GAT (v1.3.5)^74^. Considered metrics included the count of repeats intersecting with the inversion breakpoint regions and the percentage of base pairs in breakpoint regions associated with specific repeats. To search for evidence of repeat-mediated inversions, we intersected inversion breakpoint regions with TE and SD annotations. Using the 500-bp regions flanking each inversion, we called repeat-mediated inversions based on the presence of TEs from the same family at both breakpoints, or flanking SDs at both breakpoints. To investigate the relationship between inversion length and associated SD length, we performed a linear regression comparing inversion length to mean adjacent SD length, finding a significant correlation (Kendall’s *τ* = 0.63, *P* = 0.0007). To visualize SD-enriched inversion breakpoints, we generated self-versus-self alignments of breakpoint regions using minimap2 with the flag -P and produced dotplots using Python.

### Reporting summary

Further information on research design is available in the Nature Portfolio Reporting Summary linked to this article.

## Online content

Any methods, additional references, Nature Portfolio reporting summaries, source data, extended data, supplementary information, acknowledgements, peer review information; details of author contributions and competing interests; and statements of data and code availability are available at 10.1038/s41588-025-02367-z.

## Supplementary information

Supplementary Information Supplementary Notes 1–4 and Supplementary Figs. 1–6.

Reporting Summary

Supplementary Tables 1–21 Supplementary Tables 1–21.

## Figures and Tables

**Extended Data Fig. 1:**
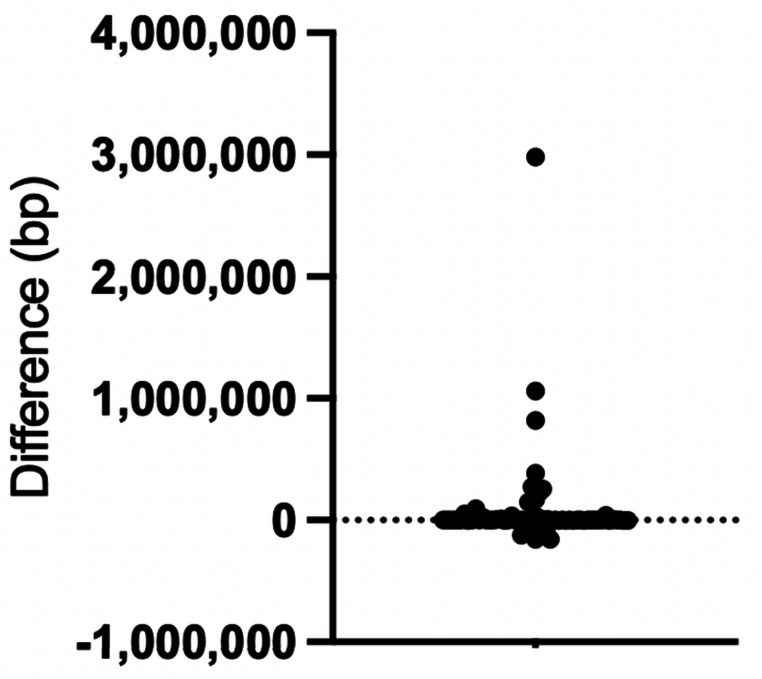
Gap sizes in GRCm39 vs. T2T C57BL/6J. Difference between estimated gap size in GRCm39 and gap-filling sequence size in T2T C57BL/6J (n = 70). Data are presented as median values with interquartile range (IQR).

**Extended Data Fig. 2:**
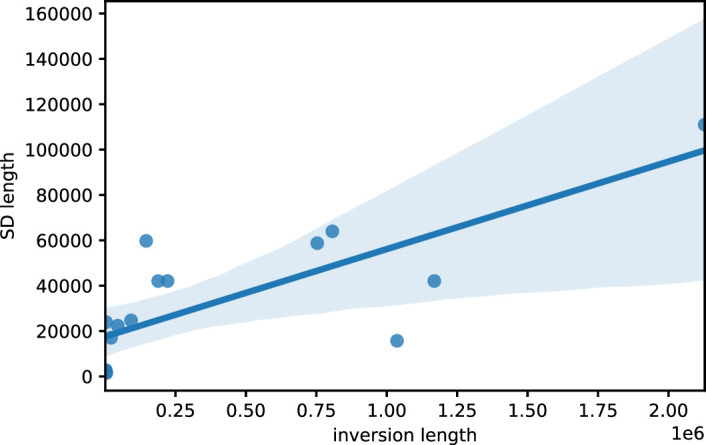
Inversion length vs. Flanking segmental duplication length. Scatter plot showing inversion length and mean associated SD length for each inversion. The regression line represents a robust linear model (RLM; linear model that is robust to outlier effects) and the shaded area denotes 95% confidence intervals. Inversion length and mean associated SD length are significantly correlated (n = 16, Kendall’s tau =0.628, p = 0.000725).

## Data Availability

The genome sequencing reads and assemblies are available from the European Nucleotide Archive under BioProject PRJEB47108, with assembly accessions GCA_964188535 (C57BL/6J) and GCA_964188545 (CAST/EiJ). The genome assemblies and annotation are available via the Ensembl (https://projects.ensembl.org/mouse_genomes/) and the UCSC Genome Browsers. The Genbank accession for the C57BL/6J PAR sequence is BR001762.

## Code availability

No custom code package or newly developed algorithm was generated in this study. All analyses were performed using default settings unless otherwise described in the methods.
