# Supplementary material for: Complete genome assemblies of two mouse subspecies reveal structural diversity of telomeres and centromeres
Source: Nat Genet. 2025 Oct 21;57(11):2852–62. doi: 10.1038/s41588-025-02367-z (PMC12597820; doi:10.1038/s41588-025-02367-z)
Supplement: Supplementary file 1 — Supplementary Notes 1–4 and Supplementary Figs. 1–6. [file 41588_2025_2367_MOESM1_ESM.pdf]

nature genetics

Supplementary information

https://doi.org/10.1038/s41588-025-02367-z

Complete genome assemblies of two mouse subspecies reveal structural diversity of telomeres and centromeres

In the format provided by the authors and unedited

Supplementary Note 1

Significant deviations in mapped reads are observed on chromosomes 2 and 9 due to the presence of short satellite-like sequences within two regions of these chromosomes. In this study, the sex chromosomes for C57BL/6J were not assembled to T2T quality and remain to be completed. Complete BUSCO genes in both genomes are 99.6% (C57BL/6J) and 99.2% (CAST/EiJ) (Supplementary Table 3).

To compare the accuracy of the C57BL/6J T2T assembly and the GRCm39 reference genome, we measured concordance of both against Pacbio reads from the C57BL/6J 'Eve' mouse by calling structural variants as putative errors and misassemblies [1]. 11% fewer structural differences were predicted from the T2T genome compared to GRCm39 - insertions (4,147 vs 4,496), deletions (3,192 vs 3,295), duplications (0 vs 3), and inversions (660 vs 654) (Supplementary Table 4).

Supplementary Note 2

We measured accuracy of the strain assignment of centromere in the genomes by aligning pure ONT reads from mhaESC (C57BL/6) to both our T2T genomes and find that 0.5% (1.58 Mbp) of the CAST/EiJ centromere regions have mhaESC read coverage (Supplementary Table 11, see methods). We used read coverage to determine if the centromere regions contain misassembled regions (e.g. incorrect repeat copy number). We realigned our T2T reads from the F1 to both assemblies and find that the majority (99.6% in C57BL/6J and 99.7% in CAST/EiJ) of the centromere regions have read coverage less than twice the sequencing coverage (Supplementary Table 12).

Supplementary Note 3

We located a large, tandem SD present between the *Mid1* and *Mafl* genes in both strains. The repeat unit size is approximately 85 Kbp in C57BL/6J and 72 Kbp in CAST/EiJ, and a copy number polymorphism is observed even in inbred mice. The copy number was quantified using digital PCR in 10 C57BL/6J mice purchased from the Jackson Laboratory, and a range of copy numbers per diploid was detected, with values between 2 and 23 (on average, 10.5). A comparison of the two strains revealed not only disparate locations of the PAR boundary, different sizes of the SD, and divergent physical sizes of the remaining region, but also a multitude of amino acid substitution mutations in all PAR genes (on average, one mutation per approximately 19 amino acid residues). The mouse PAR is a unique region of the mouse genome, exhibiting variation consistent with rapid change in sequence and structure.

Supplementary Note 4

For example, over 48 novel putative KZFPs in the C57BL/6J T2T genome have been identified. We observed large scale structural variations in KZFP clusters and significant

divergence in the number of putative KZFPs between the two mouse strains (Fig. 5a and b). The chromosome 2 cluster in CAST/EiJ contains over 1.5 Mbp of additional sequence and an additional 42 putative KZFPs compared to the C57BL/6J genome (Fig. 5a). These additional genomic sequences have high homology to the elements present in the C57BL/6J genome, suggesting that they arose as the result of duplication events. The chromosome 4 KZFP cluster has a 2 Mbp inversion in the DNA sequence, as well as multiple potential SDs between the C57BL/6J and CAST/EiJ genome assemblies (Fig. 5b). However, other KZFPs clusters, such as the KZFP cluster on chromosome 17, do not show a significant improvement in sequence annotation using the T2T assemblies and do not show the same levels of polymorphism between mouse strains (Fig. 5c), indicating the distal KZFP clusters on chromosomes 2 and 4 are more highly variable and may be under additional evolutionary pressure. Bruno *et al.* recently extended this KZFP comparative analysis to include 129S1/SvImJ, identifying significant heterogeneity of three young KZFP gene clusters and hypothesize that the varying numbers of copies of the same KZFP across different mouse strains could also influence transcriptional repression[2].

References

1. Sarsani, V. K. *et al.* The genome of C57BL/6J ‘Eve’, the mother of the laboratory mouse genome reference strain. *G3 (Bethesda)* **9**, 1795–1805 (2019).
2. Bruno, M. *et al.* Young KRAB-zinc finger gene clusters are highly dynamic incubators of ERV-driven genetic heterogeneity in mice. *Genetics* (2025).

C57BL/6J

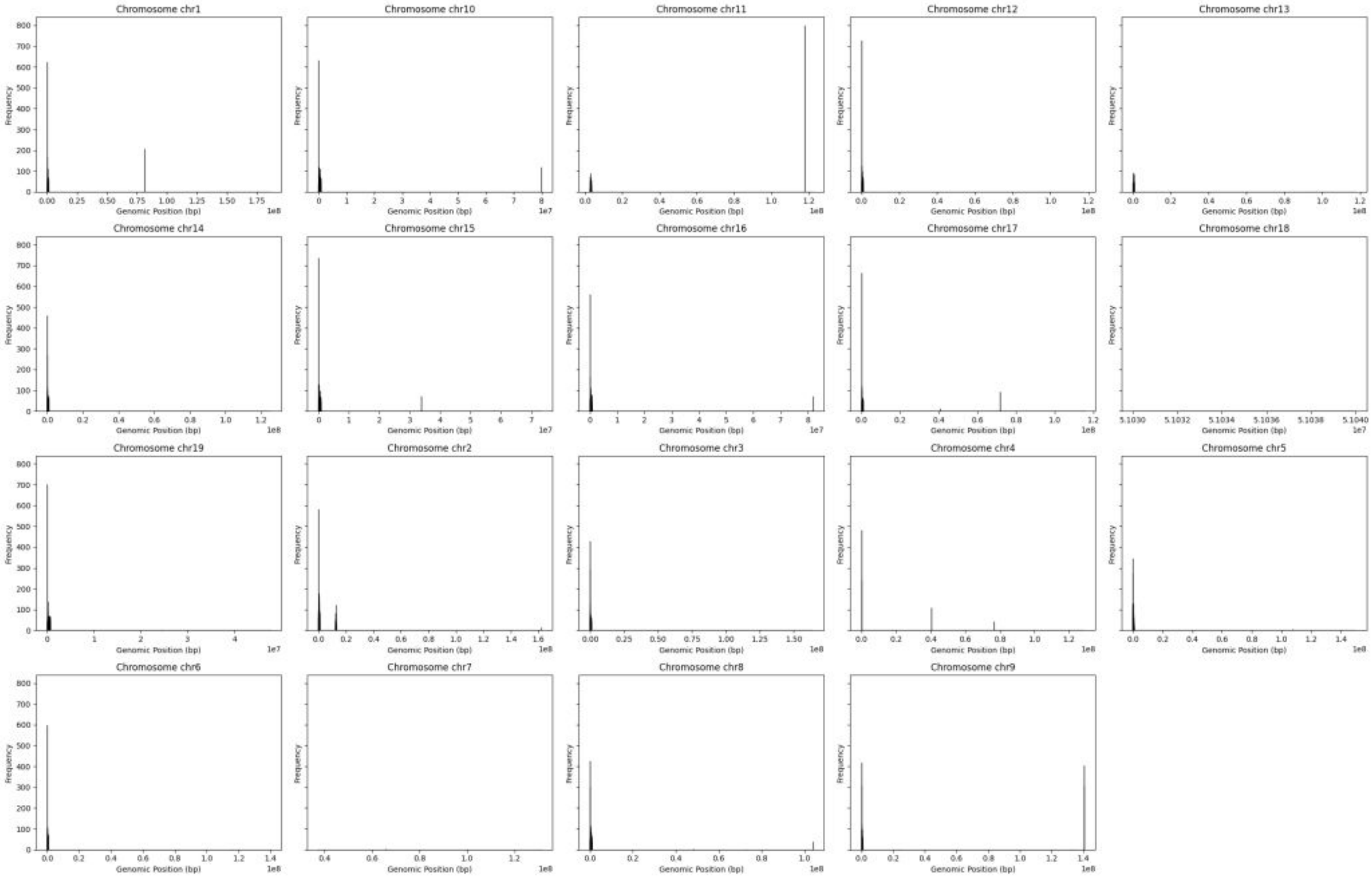

CAST/EiJ

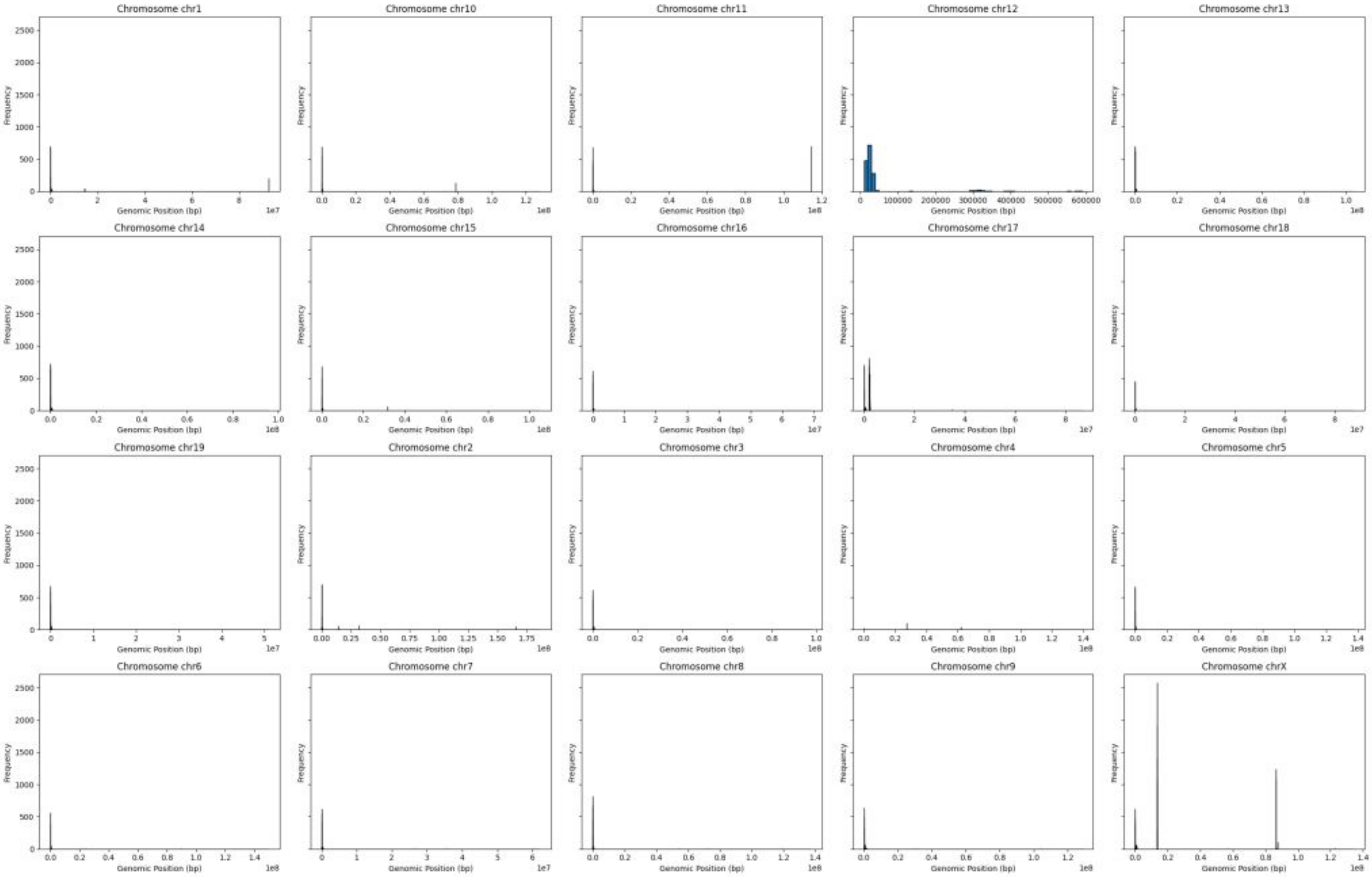

Supplementary Figure 1: Location of TLC sequences in C57BL/6J and CAST/EiJ autosomes

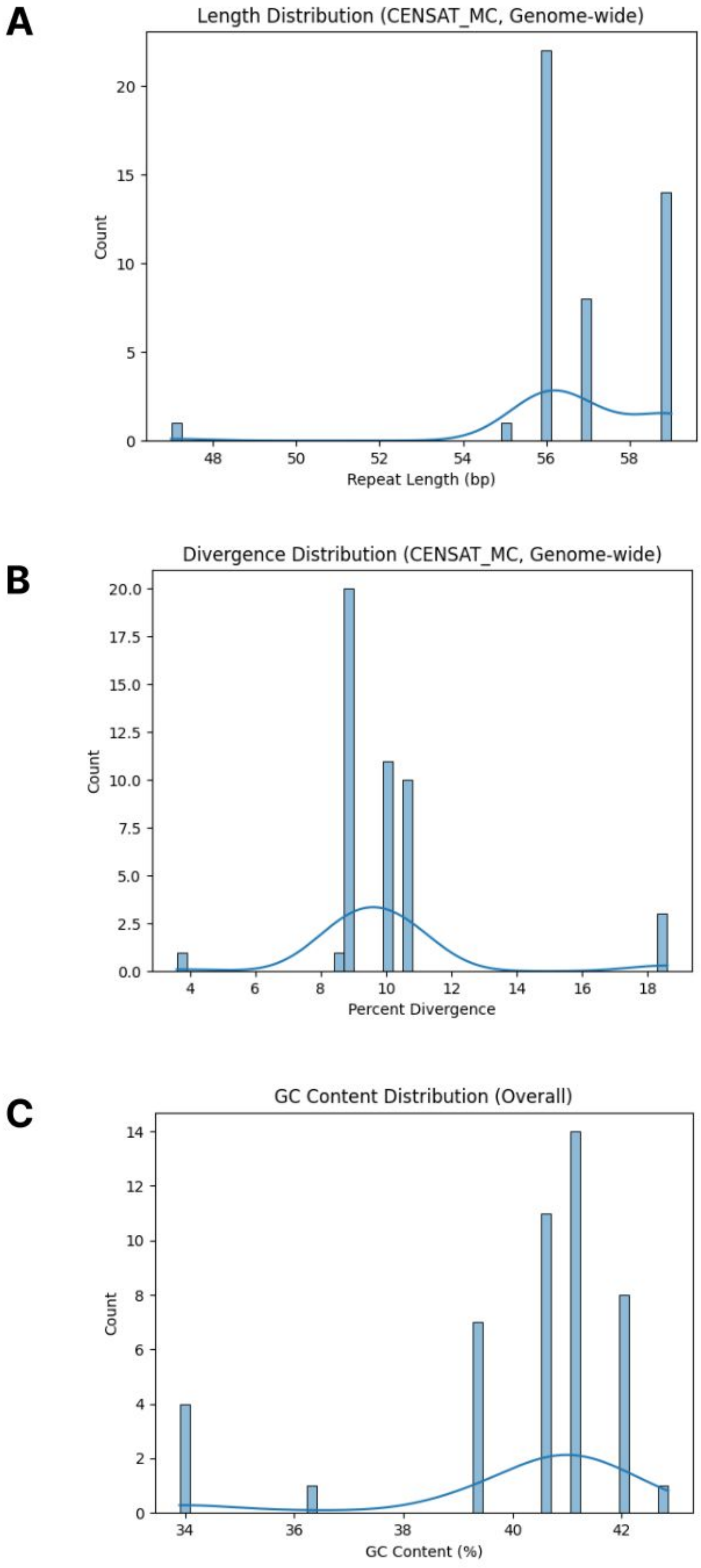

Supplementary Figure 2: Genome-wide characterisation of CenSat repeat elements in CAST/EiJ. (A) Histogram and kernel density estimate (KDE) of CenSat repeat lengths (B) Histogram and KDE of RepeatMasker percent sequence divergence for each CenSat insertion from its consensus sequence. Divergence values are computed by RepeatMasker as the Kimura two-parameter–corrected proportion of mismatched bases in the alignment, normalized by insertion length (e.g., 10% indicates ~10 mismatches per 100 bp after correction). © Histogram and KDE of GC content (%) for CenSet sequences

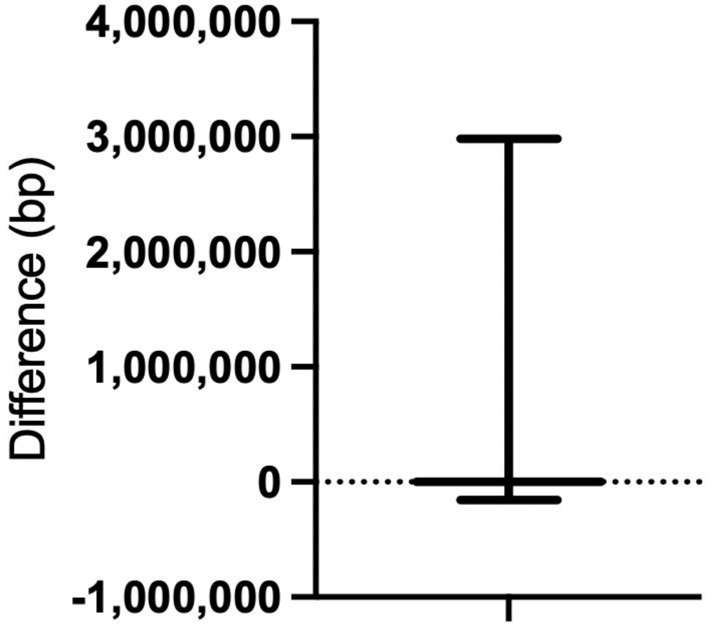

Supplementary Figure 3: Difference between estimated gap size in GRCm39 and gap-filling sequence size in T2T C57BL/6J

A

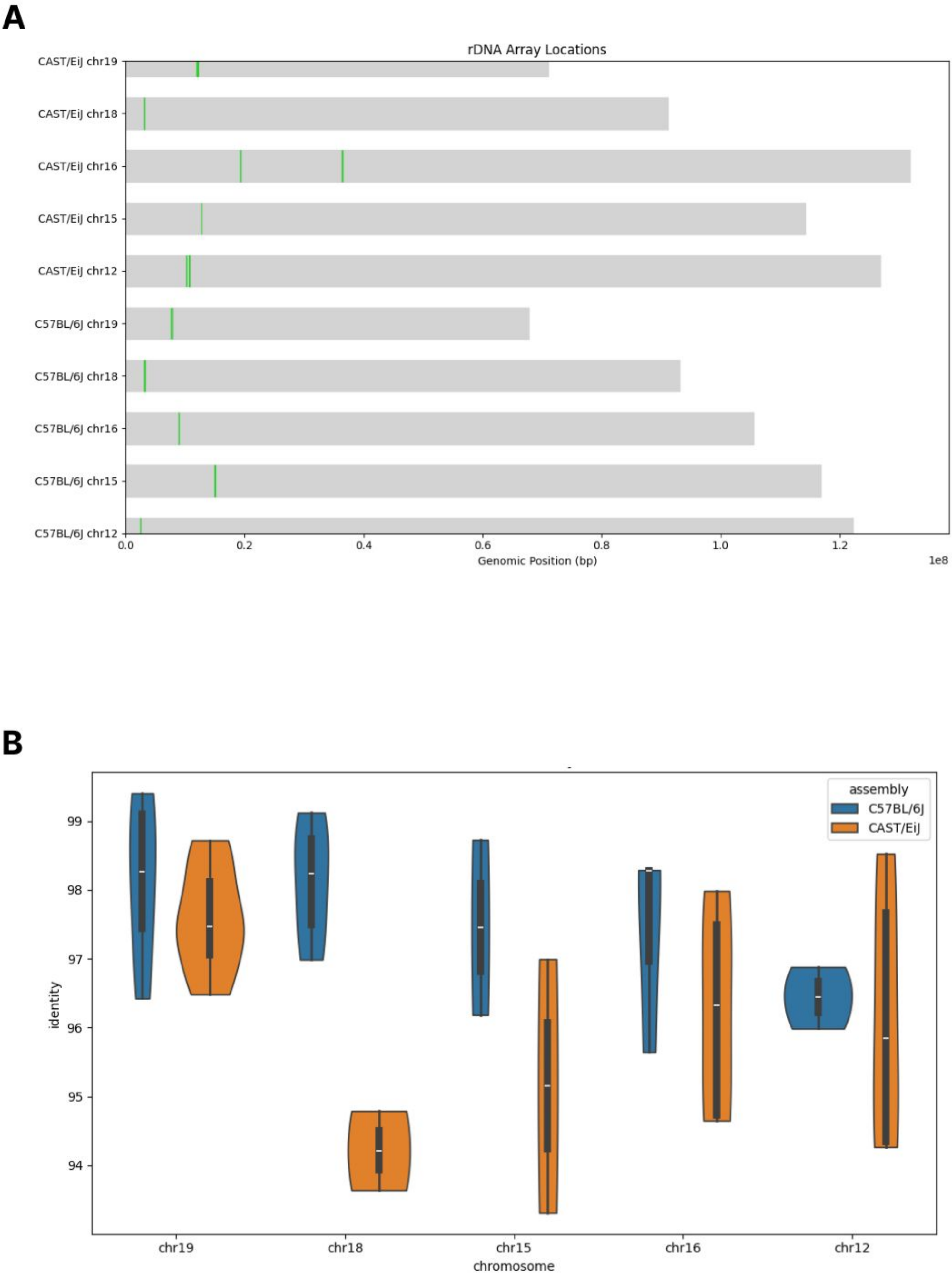

Supplementary Figure 4: (A) Location of the rDNA arrays on the chromosomes in both strains. (B) Distribution of the BLAST % identity hits for the complete mouse rDNA repeating unit by both chromosome and strain

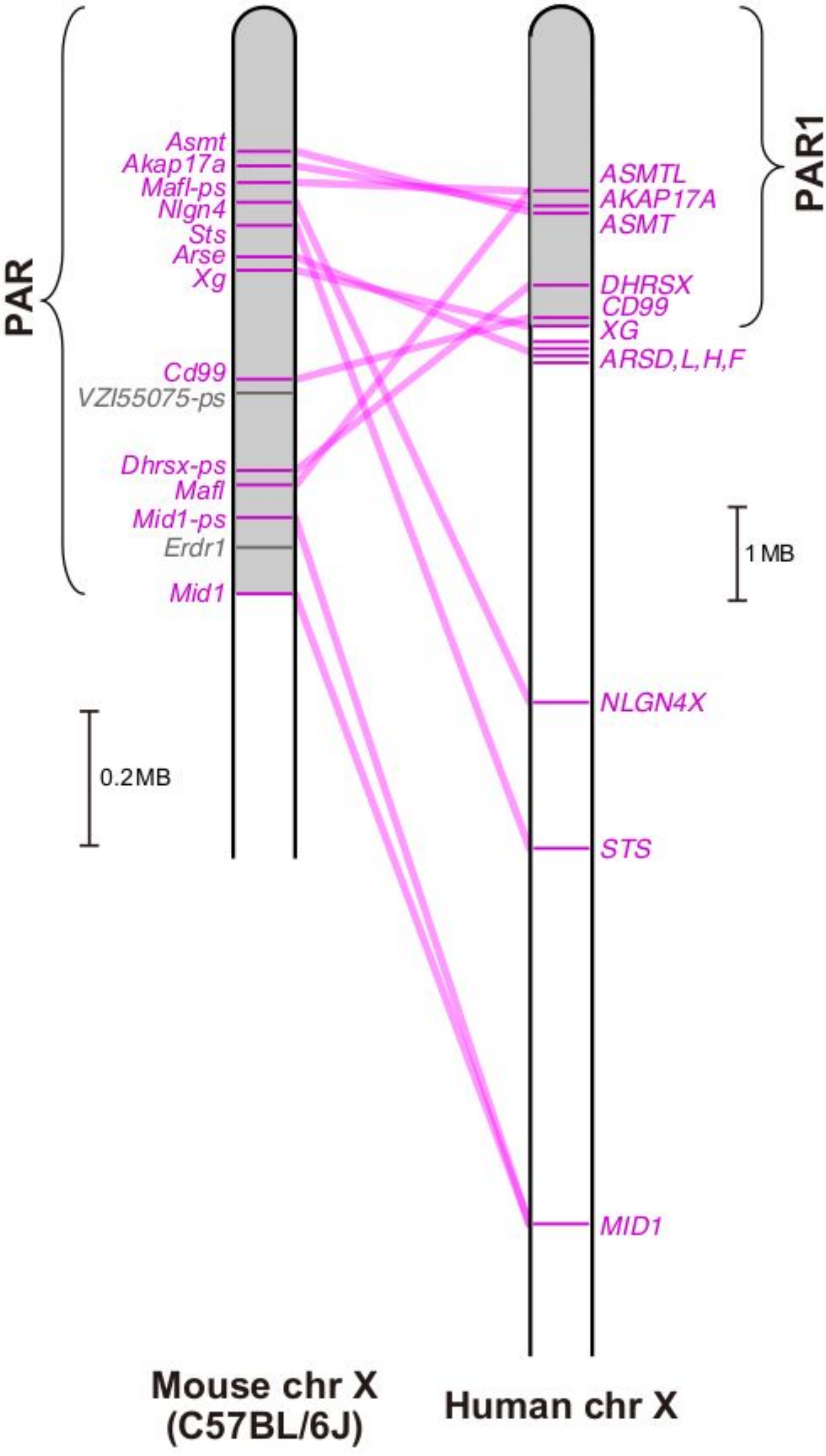

Supplementary Figure 5: Comparison of mouse PAR genes and human PAR1 genes. Orthologous pairs of human genes located in the PAR1 or the PAB region of the X chromosome and mouse genes are connected by lines. Figure derived from Kasahara et al. (2022).

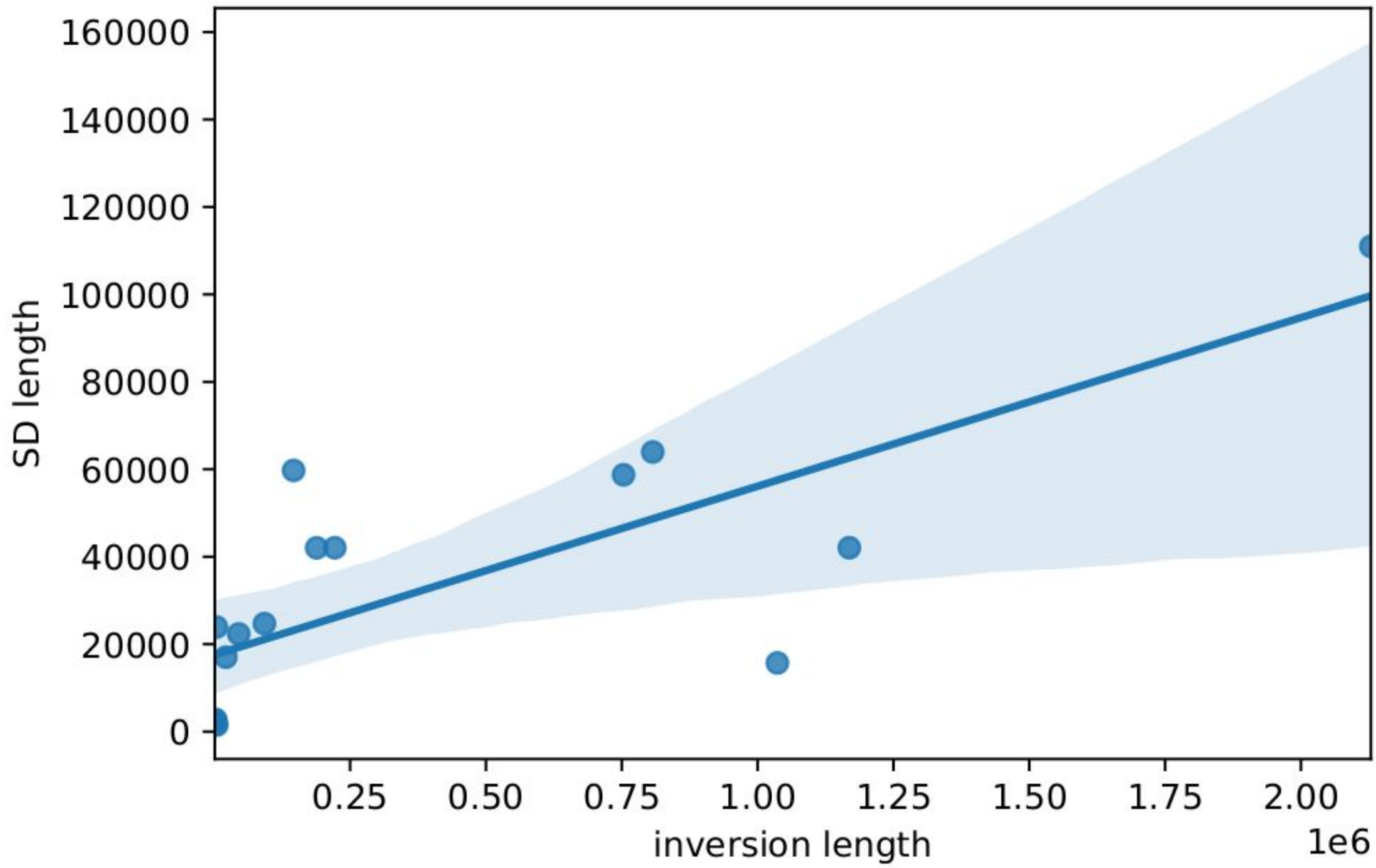

Supplementary Figure 6: Inversion length vs. flanking segmental duplication length. Larger SDs are associated with larger inversions.
